# More Than Effects in Skin: Ultraviolet Radiation-Induced Changes in Immune Cells in Human Blood

**DOI:** 10.3389/fimmu.2021.694086

**Published:** 2021-06-10

**Authors:** Prue H. Hart, Mary Norval

**Affiliations:** ^1^ Telethon Kids Institute, University of Western Australia, Perth, WA, Australia; ^2^ Biomedical Sciences, University of Edinburgh Medical School, Edinburgh, United Kingdom

**Keywords:** immunosuppression, sunlight, phototherapy, T cells, B cells, T regulatory cells, natural killer cells, dendritic cells

## Abstract

Cells of the skin and circulation are in constant two-way communication. Following exposure of humans to sunlight or to phototherapy, there are alterations in the number, phenotype and function of circulating blood cells. In this review, only data obtained from human studies are considered, with changes induced by UV radiation (UVR) exposure described for phagocytic leukocytes and peripheral blood mononuclear cells plus their component T and B cells, natural killer cells and dendritic cells. These immune modulations illustrate the potential of UVR to have therapeutic effects beyond the skin, and that sunlight exposure is an important environmental influence on human health.

## What’s already known about this topic?

- Skin exposure to ultraviolet radiation is immunosuppressive and is used to treat inflammatory skin conditions
- No previous review of changes in human blood cells after natural sun exposure or phototherapy

## What does this study add?

- Collates reports of changes to the number, phenotype and function of blood cells in humans following natural and experimental ultraviolet radiation exposure
- Supports proposals that UVB phototherapy alters several blood cell types that may have biological consequences in health and disease

## Introduction

At the surface of the earth, ultraviolet radiation (UVR) from the sun contains approximately 6% UVB (waveband 280-315 nm) and the remaining 94% is UVA (waveband 315-400 nm: divided into UVAI 340-400 nm and UVAII 315-340 nm). The exact proportions of terrestrial UVB and UVA and the total ambient dose depend on the latitude, season, time of day, cloud cover, air pollution and surface reflection, while, in addition, personal sun exposure depends on the area of the body irradiated. Due to these variable factors and to difficulties in accurately assessing past sun exposure in people, artificial UV sources are most frequently used to monitor the immunological effects of UVR in humans. Lamps emitting solar simulating radiation (SSR) have been developed which mirror the solar UVB and UVAII wavebands, and those emitting narrowband (NB)-UVB (peak at 311 nm), broadband (BB)-UVB (280-350 nm), UVA (315-400 nm) and UVAI (peak 368 nm) are also available [reviewed in (1)].

Exposure to solar UVR and to artificial UV sources has both beneficial and harmful effects on human health. These can be direct such as by causing mutations in the irradiated skin. They can also be more indirect, acting not only in the irradiated body site but also systemically [reviewed in (2)]. UVB penetrates only as far as the epidermis with UVA penetrating more deeply into the dermis. Following cutaneous irradiation, several chromophores have been identified that initiate anti-inflammatory and immunosuppressive mechanisms locally, and also drive a cascade of immune changes systemically [reviewed in (2–4)]. These responses are mediated by a variety of factors including nitric oxide, *cis-*urocanic acid (*cis*-UCA), ligands of the arylhydrocarbon receptor, platelet activating factor, prostaglandin E2, antimicrobial peptides and the synthesis of vitamin D from epidermal 7-dehydrocholesterol. Thereafter, it is proposed that there may be cellular changes in the lymph nodes draining the irradiated site, haematopoietic alterations in bone marrow, and possible modulations in circulating blood cells, perhaps engineered by cytokines and other immune mediators released initially from the irradiated skin site.

The majority of studies on immunomodulation in humans following solar or artificial UVR exposure examine effects in the skin or in a particular organ. In this Mini Review, the aim is to draw together the evidence that there are important UVR-induced changes in the number, phenotype and function of circulating blood cells. The sections below deal in turn with phagocytic leukocytes and peripheral blood mononuclear cells (PBMCs) including details on T and B cells, natural killer (NK) cells and dendritic cells (DCs). Only data obtained from human studies are included, as recent findings suggest that some immune outcomes in UVR-exposed human skin are not the same as those reported in mice (5). It should be noted that UVR-induced changes in blood immune cells might differ between healthy people, those responding to vaccination or infection, and patients with diseases treated by phototherapy.

## Effects of UVR on Phagocytic Leukocytes in Blood

The main cell types involved here are monocytes/macrophages and neutrophils which are essential components of the innate immune system. When healthy subjects were exposed to repeated whole body suberythemal UVB irradiation for four weeks, both the phagocytic and chemotactic activity of neutrophils was decreased, with an associated reduction in adhesion, important for binding of neutrophils to endothelial cells (6). Furthermore, following a single whole body exposure of healthy individuals to one minimal perceptible erythemal dose of UVB (max 313 nm), the adhesion and phagocytic abilities of phagocytic cells were reduced by 50% (7). This was explained by a decrease in the expression of several complement receptors and IgG Fc receptors, including CD16 (FcγRIII), which are required for adhesion and phagocytosis.

However, when seven suberythemal whole-body UVB exposures were given within 14 days, there was a significant increase in CD16 expression on monocytes (7). Following NB-UVB therapy of patients with multiple sclerosis (MS), three times weekly for eight weeks, there was no change in the overall frequency of monocytes as a percentage of total PBMCs (8). During the phototherapy, there was a lower frequency of classical monocytes (CD14+CD16-) and a higher frequency of intermediate monocytes (CD14+CD16Int) implying that the expression of CD16 may have increased (8). Intermediate monocytes by increased IL-10 production (9) may be associated with the immunosuppressive effects of UVR exposure and supports the anti-inflammatory properties of UVR on phagocytic leukocytes that may be relevant during infections.

## Effects of UVR on PBMCs

Two possible adverse effects of UVR on PBMCs, namely apoptosis and altered proliferative ability, have been examined in limited studies.

Apoptosis was monitored in healthy volunteers following whole body UVB or SSR with 0.7 personal minimal erythema dose (MED) on 10 consecutive days (10). The UVB exposure was equivalent to around 35 minutes outside on a clear sky day in mid-Europe around midday, and the SSR to 15 minutes under the same conditions. Apoptosis was assessed by DNA fragmentation 24 hours after the final irradiation. It was enhanced in the PBMCs from the irradiated individuals, with SSR being more effective than UVB, possibly because the UVA emitted by the SSR can penetrate more effectively into the dermal microvasculature. In addition, SSR had a greater effect on reducing the expression of anti-apoptotic proteins while increasing that of pro-apoptotic proteins, leading to the suggestion that UVA induces apoptosis of lymphocytes via photosensitised oxygen radicals (10).

Results from testing the effect of UVR on the ability of PBMCs to proliferate *in vitro* in response to antigen-specific stimuli and mitogens have varied, perhaps due to the use of lamps emitting different spectra and the range of *in vitro* stimuli (11, 12), although the majority suggest no effect. Gilmour et al. (13) monitored patients with psoriasis, some of whom had latent herpes simplex virus (HSV), following standard BB-UVB phototherapy. There was no change in the lymphoproliferative response to a T cell mitogen, or to HSV antigen throughout the phototherapy. However, there was a substantial reduction in the ability of epidermal cells prepared from the irradiated skin to present HSV antigens. Similarly, whole body suberythemal irradiation with SSR of healthy volunteers on five consecutive days had no effect on the subsequent ability of their PBMCs to proliferate in response to mitogens or to the recall antigens, diphtheria and tetanus toxoid (14). Also, the lymphoproliferative response to mitogens in healthy subjects was unchanged following exposure to suberythemal UVB radiation on five consecutive days in another study (15). In contrast, whole body NB-UVB of patients, the majority with psoriasis, for four weeks during winter led to a reduction in their lymphoproliferative response to anti-CD3/CD28, a T cell stimulant, as well as a decrease in IL-10 production (16).

Any altered function of circulating cells may reflect UVR-associated epigenetic changes to genes causing altered transcription of those genes. In one study of DNA in circulating lymphocytes, increasing solar UV exposure of individuals reduced the methylation of DNA repeat sequences, such as the long-interspersed nucleotide elements that are generally heavily methylated to silence their expression (17). Bustamante et al. (18) examined transcriptional changes in PBMCs in nine healthy volunteers pre- and 6, 24 and 48 hours post-exposure to approximately one MED of SSR. Transcripts of multiple genes associated with wide-reaching health effects were downregulated. In both studies, changes were independent of plasma levels of 25-hydroxyvitamin D, used as a marker of vitamin D status. However, others have found that vitamin D status and vitamin D_3_ supplementation can alter the transcription of hundreds of genes in white blood cells, thus suggesting widespread changes in demethylation of genes with changing vitamin D levels (19).

## Effects of UVR on T Cells in Blood

The changes in PBMCs induced by UVR described above are likely to reflect principally modulations in T and B cells as T cells comprise 60-80% of PBMCs and B cells 5-15%. In **Table 1** the effects of UVR on multiple subsets of circulating T cells are shown, principally identified by expression of surface and intracellular markers, and lineage-specific transcription factors, such as Foxp3.

**Table 1:** Changes to circulating T cell subsets associated with UVR exposure. T cells express CD3, the T cell co-receptor. They are then subdivided according to their phenotype that frequently reflects their function. Classification has often been aided by detection of intracellular cytokine expression after a short stimulation *in vitro*. CXCR5 allows cell migration into germinal centres.

Cell subset	Phenotype	% of CD3+ cells*	% CD4+ cells*	Changes after UVR exposure
T helper (Th)	CD3+CD4+	60-70		No change (8)
Th1	CD3+CD4+CXCR3+ (T-bet+) making IFNγ on in vitro stimulation		variable	No change (20) Reduced (21)
Th2	CD3+CD4+CXCR3-CCR6-, making IL-4 on in vitro stimulation		variable	No change (20) Reduced (21)
Th17	CD3+CD4+CXCR3-CCR6+ (ROR-γt+) making IL-17 on in vitro stimulation		variable	Increased (20) No change (8) Decreased (21, 22)
Skin-homing Th	CD3+CD4+CLA+CCR4+ (ref)		variable	Decreased (21)
Treg	CD3+CD4+CD25+Foxp3+(sometimes CD27+/CD127lo)		5	No change as % of T cells (8, 20, 23, 24) Increased as % of T cells (16, 25, 26) Increased activated Treg implied by activation-associated phenotype (23) Increased Treg function (25, 26)
T follicular helper	CD3+CD4+CXCR5+		15	No change (8)
T follicular regulatory	CD3+CD4+CXCR5+Foxp3+		1.5	No change (8)
T cytotoxic	CD3+CD8+	20-30		No change (8, 27, 28)

*Percentages in healthy individuals (8).

### T Cell Changes Associated With Natural Sunlight Exposure

When 217 healthy adults from Townsville (latitude 29^o^S) and Canberra (latitude 35^o^S), Australia, were immunized with an experimental T-cell-dependent antigen, keyhole limpet hemocyanin, a delayed-type hypersensitivity response to antigen recall challenge 21 days after immunisation was lower in individuals with higher personal clothing-adjusted UVR exposure on the day before immunisation and during intervals spanning the day before to 2–3 days after immunisation (20). Higher personal UVR exposure was associated with a small incremental increase in blood Th17 cells (as a proportion of CD4^+^ T cells) from pre- to post-immunisation, but no changes in the numbers of Th1 or Th2 effector Tregulatory cells (Treg) occurred (20).

Twenty patients with psoriasis were subjected to controlled sun exposure daily for 16 days on Gran Canaria, Canary Island, Spain, resulting in a persistent selective reduction of skin-homing cutaneous lymphocyte-associated antigen (CLA)+ T cells in blood, first apparent after only one day in the sun (21). At 16 days, the PBMCs demonstrated a reduced capacity to secrete IFN-γ, IL-17, TNF-α, and IL-10 compared with baseline levels, suggesting systemic immunosuppression.

When skin pigmentation readings obtained by spectrophotometry were used as a marker of recent UVR exposure, there were no correlations between UVR exposure and the overall proportion of circulating PBMCs, and with Treg subsets defined by CD45RA, CD27, FoxP3 and CD25, in the blood of 350 individuals undergoing routine skin cancer screening (23). However, Tregs with an activation-associated phenotype, CD45RA-/CD27-, and those expressing skin homing receptors (CLA, CCR4), were positively associated with recent UVR exposure, particularly among lighter-skinned participants, suggesting potential enhanced Treg activity.

### T Cell Changes Associated With NB-UVB Phototherapy

There have been several reports of increased numbers in blood of CD4+CD25+FoxP3+ Tregs following NB-UVB phototherapy of patients with psoriasis (22, 25) and polymorphic light eruption (26), with increased regulatory ability in some instances (**Table 1**). Cell changes in the blood of patients with MS have also been followed after experimental NB-UVB three times per week for six (24) or eight weeks (8, 29) with no increases detected in functional Tregs in those receiving NB-UVB phototherapy. In the first study of nine patients, although no change in the percentage of blood CD4+CD25+CD127lo Tregs occurred, the percentage of Helios-negative cells within that subset was higher at the time of UVB cessation. Without functional studies, this finding was of little meaning as Helios expression may be a marker of Treg activation (24) or represent Tregs with unknown regulatory function (30, 31). In the second study, when PBMCs from ten irradiated patients were compared with the same number, also with MS, who did not receive the intervention, there was no association of phototherapy with the frequency of CD4+ or CD8+ T cells, Tregs or T follicular regulatory cells as a percentage of PBMCs, irrespective of whether Tregs were defined as CD4+FoxP3+, CD4+CD25+CD127lo or expressed differing levels of Helios (8) (**Table 1**).

To conclude, some reports, although not all, support a UVR-induced increase in the number and function of Tregs (**Table 1**). If recruited back to irradiated skin or more distal tissues, Tregs may potentially assist in controlling inflammatory and autoimmune pathways.

## Effects of UVR on B Cells in Blood

There is little evidence that UVR exposure alters antibody production. In the study described above (20), keyhole limpet hemocyanin-specific IgG1 and IgG2 titres were not associated with acute or cumulative UVR exposure. When healthy volunteers who were whole-body irradiated with one MED of SSR on five consecutive days and then vaccinated intramuscularly with recombinant hepatitis B surface antigen, no effect of the radiation on antibody responses was detected compared with unirradiated controls (14). In a further study (32), SSR (1.3 standard erythema dose, three times per week for four weeks beginning three days after the initial vaccination) did not influence hepatitis vaccination efficacy as assessed by serum antibody titres.

However, B cells play major roles in human diseases, not only as antibody-secreting cells but also as antigen presenting cells and cytokine producers, with particular emphasis in autoimmune diseases such as MS (33). Trend et al. (8) demonstrated that, in patients with MS, the most substantial short-term changes in lymphocyte subsets during NB-UVB phototherapy involved B cells. In particular, compared with unirradiated patients, a decrease in memory B cells and an increase in naïve B cells, both as a percentage of B cells, occurred. This was complemented after two months of phototherapy by a reduction in IgG3+ B cells as a percentage of B cells in blood (34). Furthermore, the *in vitro* functional responses of memory B cell subsets for production of the pro-inflammatory cytokine, tumour necrosis factor, was reduced at the end of the phototherapy (35). These results suggest that NB-UVB may have potential to reduce the pathology of B-cell driven immune conditions by reducing inflammatory cell-cell-communication and inflammatory cytokine production, possibly involving the induction of type I IFN and its associated pathways (36).

## Effects of UVR on NK Cells in Blood

Of the circulating lymphocytes, 5-15% are NK cells. They are important players in the innate immune system as they are major histocompatibility-unrestricted and used in the recognition and lysis of virally infected cells and tumour cells. Due to their production of IFN-γ, they also promote the development of Th1 immune responses.

There is very good evidence that exposure to UVR substantially suppresses NK cell cytotoxicity. Gilmour et al. (27) reported that there was a decline in NK cell activity following treatment of psoriasis patients with BB-UVB or NB-UVB phototherapy over a period of six weeks. It took several weeks thereafter to return to the pre-treatment level of activity. When healthy volunteers were whole-body irradiated with BB-UVB using the same protocol as employed in the treatment of psoriasis, NK cell activity was reduced in all subjects after 10 days and declined more as the irradiations progressed; recovery to pre-irradiation levels took seven days (28). Similarly, Sleijffers et al. (14) reported suppression in NK cell activity following whole body UVB irradiation of healthy volunteers on five consecutive days. Only one study has monitored numbers of NK cells in blood during UVR exposure. Trend et al. (8) found that there was no change in the frequency of either mature or immature NK cells as a percentage of the PBMC following exposure of MS patients with NB-UVB three times weekly for eight weeks; five sub-populations of NK cells were examined according to their expression of CD56, CD16 and CD57.

The mechanism whereby UVR suppresses NK cell activity without altering their number in blood is unknown but may be due to the UVR-induced alterations in the release of soluble mediators into the blood, such as *cis-*UCA (27), or to reduced production of IL-12 which is required to stimulate NK cells (37). Reduced NK cell activity after UVR exposure may contribute to UVR-induced skin cancer development.

## Effects of UVR on DCs in Blood

DCs in blood represent less than 1% of PBMCs and are dissimilar from tissue DCs as they have no dendritic processes and do not express maturation markers such as CD83. They are separated by phenotype and function into three types: plasmacytoid DCs (pDCs) which recognise viral antigens and produce type 1 IFNs, and two subtypes of myeloid DCs (mDCs), one CD1c+ and the less frequent CD141+, which are highly phagocytic and process both viral and bacterial antigens.

Limited experiments have assessed the effect of UVR exposure on blood DCs. In summary, whole body irradiation of healthy individuals with suberythemal doses daily for up to 30 days and subsequent phenotypic analysis of PBMCs revealed no change in the percentage of pDCs with a very small increase in the percentage of CD1c+ mDCs in one study [UV source 4% UVB/96% UVA (38)] and a similarly small increase in the percentage of CD141+ mDCs in a second study [UV source 54% UVB/46% UVA (39)]. Different UV spectra could account for the different results. DCs have also been assessed in the blood of patients with MS following NB-UVB phototherapy (8). No change in the frequency of DCs as a percentage of the total PBMCs or of the three DC subsets (CD141+ myeloid, CD1c+ myeloid and CD303+ pDCs) was detected compared with unirradiated control patients. Thus, despite the sparse evidence, it seems unlikely that exposure to UVR affects the number of circulating DCs or subsets significantly. Functional and migratory tests on blood DCs have not been carried out thus far although murine tests suggest their function may be reduced (40–42).

## Seasonal Effects on Immune Cell Profiles in Blood

In a large British healthy adult population, periodic seasonal changes in the total number of white blood cells, as well as lymphocytes, monocytes, basophils, eosinophils, neutrophils and platelets have been reported and may reflect different challenges to the immune system in winter and summer (43). For example, winter is associated with increased monocytes and inflammation. When co-regulated seasonal mRNAs in PBMCs from a cohort of German children were analysed, genes for pro-inflammatory processes were more frequently expressed in winter, compared with summer (43). A similar 12-month seasonal cycle in immune cell subsets has been detected in the blood of 606 healthy Australian adults (44). Different lengths of the photoperiod may contribute to seasonal blood cell patterns; however, a more inflammatory immune system in winter is compatible with higher levels of immunoregulatory solar UVR in summer. In a study of patients with polymorphic light eruption in Graz, Austria (47^o^N) from winter to summer, increases in the prevalence and suppressive function of blood Tregs were detected that were independent of increases in vitamin D status (45), thus supporting the involvement of non-vitamin D-dependent, UVR-induced pathways of immunosuppression, as described in section 2 above.

## Conclusions

Phototherapy has had considerable success in controlling inflammatory skin conditions by local proapoptotic, immunomodulatory, antipruritic, antifibrotic, propigmentary and pro-prebiotic effects [reviewed in (2, 46)]. Following exposure of individuals to sunlight or phototherapy, there are also changes in several cell types in blood, indicating that UVR has immunological effects beyond the skin. Alterations in neutrophils, T cells, B cells and NK cells have been demonstrated with little evidence for DCs. Such effects are likely to promote immune homeostasis in healthy people by dampening inflammatory processes and the immunoregulatory effects may prove beneficial in the treatment of some systemic diseases, such as autoimmune diseases like MS. Consistent results were not obtained in all instances, explained perhaps by the lack of power in some studies, or the underlying health conditions of the participants, or differences in the spectra emitted by the UV sources. More research is required to define, in particular, the association of UVR with T and B cell subsets in both healthy individuals and patients with various inflammatory diseases, and to establish whether the UVR-induced changes in the circulating cells are due to the release of particular soluble mediators into the blood, as well as determining antigen specificity where appropriate.

## Author Contributions

The authors made equal contributions to the design and writing of the Mini Review, and approved it for publication. All authors contributed to the article and approved the submitted version.

## Funding

PHH is supported by MS Western Australia.

## Conflict of Interest

The authors declare that the research was conducted in the absence of any commercial or financial relationships that could be construed as a potential conflict of interest.
